# Seed treatment with clothianidin induces changes in plant metabolism and alters pollinator foraging preferences

**DOI:** 10.1007/s10646-023-02720-0

**Published:** 2023-12-07

**Authors:** Björn K. Klatt, Annemarie Wurz, Lina Herbertsson, Maj Rundlöf, Glenn P. Svensson, Jürgen Kuhn, Sofie Vessling, Bernardo de La Vega, Teja Tscharntke, Yann Clough, Henrik G. Smith

**Affiliations:** 1 Centre for Environmental and Climate Science, Lund University, 223 62 Lund, Sweden; 2 Department of Biology, Lund University, 223 62 Lund, Sweden; 3 School of Business, Innovation and Sustainability, Biology & Environmental Sciences, Halmstad University, 30118 Halmstad, Sweden; 4 Department of Crop Sciences, Agroecology, University of Göttingen, 37077 Göttingen, Germany; 5 Conservation Ecology, Department of Biology, Philipps-Universität Marburg, Marburg, Germany; 6 Instituto de Biologia, Universidade Federal do Rio de Janeiro, Rio de Janeiro, Brazil

**Keywords:** Neonicotinoid insecticides, Plant-pollinator interactions, Flower preference, Plant defense metabolism, Glucosinolates

## Abstract

Neonicotinoids, systemic insecticides that are distributed into all plant tissues and protect against pests, have become a common part of crop production, but can unintentionally also affect non-target organisms, including pollinators. Such effects can be direct effects from insecticide exposure, but neonicotinoids can affect plant physiology, and effects could therefore also be indirectly mediated by changes in plant phenology, attractiveness and nutritional value. Under controlled greenhouse conditions, we tested if seed treatment with the neonicotinoid clothianidin affected oilseed rape’s production of flower resources for bees and the content of the secondary plant products glucosinolates that provide defense against herbivores. Additionally, we tested if seed treatment affected the attractiveness of oilseed rape to flower visiting bumblebees, using outdoor mesocosms. Flowers and leaves of clothianidin-treated plants had different profiles of glucosinolates compared with untreated plants. Bumblebees in mesocosms foraged slightly more on untreated plants. Neither flower timing, flower size nor the production of pollen and nectar differed between treatments, and therefore cannot explain any preference for untreated oilseed rape. We instead propose that this small but significant preference for untreated plants was related to the altered glucosinolate profile caused by clothianidin. Thereby, this study contributes to the understanding of the complex relationships between neonicotinoid-treated crops and pollinator foraging choices, by suggesting a potential mechanistic link by which insecticide treatment can affect insect behavior.

## Introduction

Systemic insecticides and in particular neonicotinoids have become an integral part of modern agriculture (Jeschke and Nauen 2008; Goulson 2013). After application, which may occur via seed coating, they translocate throughout all parts of a plant (Elbert et al. 2008). As a result, systemic insecticides provide protection against insect pests at every stage of crop growth. (Elbert et al. 2008; Jeschke and Nauen 2008). Use of neonicotinoids as a seed coating has been a particularly contentious issue for flowering crops, because of the potential exposure of pollinators via foraging on nectar and pollen (Goulson 2013; Lundin et al. 2015) and the resulting effects on fitness (Rundlöf et al. 2015; Stanley et al. 2015; Lundin et al. 2015; Woodcock et al. 2017; Siviter et al. 2021). While the outdoor use of three neonicotinoids (clothianidin, imidacloprid, and thiamethoxam) as seed-coating has been banned in the European Union to protect bees (European Parliament 2018a, 2018b, 2018c), they are still being used through emergency authorizations (European Commission 2023) as well as outside the European Union. Furthermore, other neonicotinoids are still used in the EU (acetamiprid is still approved), and recently approved insecticides, such as Flupyradifurone and Sulfoxaflor, belong to different chemical classes but have a similar mode of action as neonicotinoids by targeting the acetylcholine receptor (Zhu et al. 2011; Nauen et al. 2015). In addition, neonicotinoid residues degrade slowly and can still be found in nectar of crops several years after the ban (Wintermantel et al. 2020). Thus, there is a need to better understand the mechanism by which neonicotinoids and other systemic insecticides potentially exert effects on pollinators.

Exposure to neonicotinoids may affect plant attractiveness to pollinators, thus influencing exposure (Simon-Delso et al. 2017; Klaus et al. 2021). For example, by reducing pest pressure neonicotinoid-treated crops may be able to allocate more resources to the production of pollen and nectar, increasing their attractiveness to pollinators (Lindström et al. 2018). Defense can be costly for plants if resources are re-allocated for instance to the production of secondary compounds and hence away from plant development (Agrawal et al. 1999; Strauss et al. 2002; Kessler and Chautá 2020), resulting in, for instance, delayed flowering, decreased flower production, or a decreased production of floral resources (Agrawal et al. 1999; Strauss et al. 2002; Kessler and Chautá 2020), conseqently reducing attractiveness to pollinating insects (Stanley and Raine 2016; Kessler and Chautá 2020). Some experiments have also shown that pollinators may respond positively to the presence of neonicotinoids in sugar solution (Arce et al. 2018; Kessler et al. 2015; but see Muth et al. 2020), although the mechanism for this remains unclear. As a result, the use of neonicotinoids may influence pollinator foraging or plant visitation, thus driving their level of exposure to neonicotinoids (Simon-Delso et al. 2017; Klaus et al. 2021).

However, neonicotinoids might also indirectly influence plant attractiveness to bees. Neonicotinoids can interact with plant metabolism (Ford et al. 2010), with positive effects on plant growth and yield (Pless et al. 1971; Mitra and Raghu 1998; Macedo and de Camargo e Castro 2011; Lanka et al. 2017) but also have adverse effects on plant viability (Mitra and Raghu 1998; Szczepaniec et al. 2013; Ruckert et al. 2018). For oilseed rape, treatment with neonicotinoids results in synthesis of the stress-related phytohormon salicylic acid (Ford et al. 2010), which in Brassicaceae induces the production of glucosinolates (Kiddle et al. 1994). Glucosinolates are defense compounds, also called mustard oils, that normally increase as well as allocate in all plant tissues when Brassicaceae are attacked by herbivores or pathogens (Fahey et al. 2001) and are enzymatically hydrolyzed into toxic break-down products upon plant tissue damage, where they repel or kill many herbivores that come into contact with them (Bones and Rossiter 1996). Glucosinolates can be attractive to some insect herbivores specialized on Brassicaceae, but can also repel insects (Hopkins et al. 2009), including bumblebees (Sculfort et al. 2021). As a result, plant defense induced by neonicotinoids could also impact the attractiveness of Brassicaceae plants to pollinators.

In this study, we investigated how neonicotinoid seed coating influences the attractiveness of oilseed rape (*Brassica napus* L.) to bumblebees, potentially by affecting flowering time and the production of flowers, floral resources and glucosinolates. Oilseed rape is an ubiquitous part of agricultural landscapes (Food and Agriculture Organization of the United Nations 2021) that was (Hughes et al. 2014; Rundlöf et al. 2015), and in many places still is, regularly treated with neonicotinoid seed coating such as in Canada, one of the largest oilseed rape producers outside the European Union (Lundin 2021). Potential changes in plant attractiveness were assessed by comparing glucosinolate profiles and the production of flowers, nectar and pollen between treatments under controlled greenhouse conditions. In addition, preference by bumblebees, *Bombus terrestris* L., which are important wild bee pollinators (Fussell et al. 1992; Delaplane and Mayer 2000; Kleijn et al. 2015) was tested in outdoor cage mesocosms containing both treated and untreated oilseed rape plants.

## Methods

### Experimental plants

Oilseed rape plants (variety: Majong) were grown in a greenhouse with automated watering (regularly adjusted to the plants’ needs), temperature (day: 18 °C; night 10 °C) and light (14 h/day) in five litre pots with gardening soil (Krukväxtjord lera & kisel; Weibulls Horto). Half of the plants were grown from seeds coated with Elado® (Bayer Crop Science; 400 g/l clothianidin + 80 g/l beta-cyfluthrin) and the fungicide iprodion (Rovral®; BASF) (treated plants), and the other half was grown from seeds coated only with iprodion (Rovral®; BASF) (control plants). Elado® contains beta-cyfluthrin but this pyrethroid is not systemic (Lewis et al. 2016). In a former study also using Elado®, pollen was tested for residues of beta-cyfluthrin but it was not detected (Rundlöf et al. 2015). The prophylactic treatment with the fungicide (iprodione) was conducted to prevent differences in plant development and stress resulting from potential fungal infections in the greenhouse, which can alter glucosinolate production in oilseed rape (Li et al. 1999). Iprodione has been used for this task in studies comparing glucosinolates between oilseed rape plants before (Fieldsend and Milford 1994a, 1994b). Thereby, our treatments reflect realistic conditions in oilseed rape under real farming conditions. Seed coatings were conducted by the Rural Agricultural and Economic Society (Hushållningssällskapet) according to the recommendations of the manufacturer (Bayer Crop Science). Within each treatment, an early and a late flowering set of plants was created by sowing the seeds at an interval of 10 days, to prolong flowering to mimic fields under realistic conditions were not all plants are flowering at the same time. All plants were supplied twice with NPK fertilizer (Kristalon Blue®, Yara) and calcium fertilizer (Calcinit®, Yara) according to the recommendations of the manufacturer. These plants were used to analyse glucosinolates in plant tissues and for the mesocosm experiment.

### Glucosinolate analysis in plant tissues

For glucosinolate analysis, samples were taken from 13 of the plants grown from Elado®/Rovral®-coated seeds and from 12 of the plants grown from control seeds (only coated with Rovral®) in the greenhouse that were not moved into the mesocosms, but had the same developmental stages according to the BBCH scale (Meier 2001). At the stage of sampling, all plants were fully grown and at peak flowering according to the BBCH scale (Meier 2001). From each plant, all flowers with the pistil at a medium height of anthers (to prevent age differences) (Persson 1953), as well as all green and non-wilted leaves were cut off at the basis and collected in plastic bags. Bags were stored in the freezer (–20 °C) until use. Analyses were performed by the Max-Planck Institute for Chemical Ecology (Jena, Germany) and followed the glucosinolate extraction and analysis protocol according to Beran et al. (2014): glucosinolate profiles in plants were analyzed by HPLC (High-performance liquid chromatography), using an Agilent Technologies HP1100 Series instrument equipped with a photodiode array detector and a reversed-phase column (NUCLEODUR Sphinx RP, 250 Å~ 4.6 mm, 5-μm particle size; Macherey–Nagel, Germany). Quantification of glucosinolates was performed using the internal standard Sinalbin (4-Hydroxy Benzyl GLS).

### Flowers and flower resources

Differences in floral traits, the amount of pollen and nectar produced, floral display (width of flowers), the time from sowing until the first flower and the number of flowers produced, were assessed to capture potential differences in flower attractiveness to bees due to the treatment with Elado®. These traits were analysed from a new set of plants consisting of 6 treated and 6 control plants that were grown in the greenhouse under the same conditions as described above, from Elado® and Rovral®-treated seeds (treated) and control seeds only treated with Rovral® (control), respectively. First, the time from sowing until the opening of the first flower (start of flowering) was assessed by observing plants for open flowers every day. Flower size, nectar production, and pollen production were aimed to measure for seven flowers per plant, but for two treated plants (five and six flowers respectively) and one control plant (six flowers), a lower number of flowers in the developmental stage (mature stage, descibed below) were available. Flowers were measured in their mature state with flat crown pedals (crown petals most spread) to ensure maximum floral display, as well as nectar production and that pollen was still attached to the stamens (Persson 1953). Samples were taken during at least two different days during peak flowering to limit effects from daily variations in nectar or pollen production. Nectar was collected with a microcapillary (CAMAG, Switzerland) with a volume of one microlitre and a total length of 32.0 mm (Dungan et al. 2014). Nectar was capilated from all nectaries of a flower and the length of the nectar column was measured with a digital caliper (Pro Tools, Germany). That length was converted to volume (µl) by division with the total capillary length (32.0 mm). The amount of pollen produced is correlated to the length/size of stamens (Piotrowska 2008; Luo et al. 2009). Pollen was collected by cutting off and weighing all stamens of the flower (Ac100-A85995, Mettler AC 100, Switzerland). The width of each flower (floral display) was measured with a caliper. All of these mesurements were conducted during the same time frame to limit effects of time. From each plant, 4–7 flowers were sampled. After all plants had finished flowering, the total number of flowers was counted.

### Mesocosm experiment

The mesocosm experiment was conducted at Lund University, Sweden, in six 2*2*2 m outdoor mesocosms, covered with dark-gray fiberglass nets with a mesh size of 1.5 mm (1.3 mm aperture width) to contain bees. Mesocosms were arranged in a row with 0.5 m between mesocosms (Supplementary Material Fig. S1). An additional netted wall was installed at both sides of the mesocosm row to reduce differences between mesocosms at the inner and the outer part of the experiment (edge effects from higher exposure to weather conditions). Alltogether, 120 of the plants were transferred into the mesocosms when plants from the early set had started flowering and sorted so that each mesocosm and treatment had a similar number of plants within the same phenological stage (according to BBCH scale; Meier 2001). Within each mesocosm, 10 treated and 10 control plants were placed on one side of the mesocosm, respectively. Each treatment consisted of two rows of five plants, one for early and one for late flowering plants, to simulate natural conditions where uneven development results in protacted flower availability within a field. This setup was equal in all mesocosms. A gap of one meter was left between treatments. Within each treatment, the row of five early plants was facing the mesocosm edge and the row of five late plants was facing the gap between treatments. Possible confusing effects of the bees’ decision on flower visitation from plant-treatments of neighboring mesocosms were minimized by assigning treatments to mesocosm sides, so that neighboring mesocosm sides (distance 50 cm) had the same treatment. Except for automated watering, conditions within the mesocosms were not controlled and thus reflected ambient conditions. Draining water from the pots was captured in a ca. 15 cm broad and ca. 10 cm deep ditch and led and collected outside the mesocosms to prevent the exposure of bees (Samson-Robert et al. 2014) and control plants.

Queenright colonies of the buff-tailed bumblebee *Bombus terrestris* L. were purchased for the experiment (NATUPOL^®^; Koppert Biological Systems). Although these colonies have been confined to breeding facilities for many generations, they originate from wild populations and hence their behavior should reflect that of wild populations. Colonies were stored indoors for five days and fed with sugar solution and pollen until their placement at the mesocosms. Pollen was purchased from Biobest (Belgium), originating from honeybees being placed next to nature reserves and thereby assumed to be mostly free of pesticides, and radiated to prevent exposure to diseases. Six colonies were placed on the southern side, just outside of the six mesocosms, covered with wooden roofs on top of the colony boxes for weather protection. The number of foraging resources in the mesocosms was expected to be insufficient to support the bumblebee colonies. Therefore, the colony boxes were connected to a t-shaped, plastic tube (diameter: six centimeter) with one of the legs leading to the mesocosm with the entrance at an equal distance to treated and control plants. This allowed bumblebee workers to simultaneously access mesocosms and to forage outside (Supplementary Material Fig. S1), to access sufficient resources and to prevent over-pollination of the experimental plants. The area around the experimental setup is dominated by houses with gardens and university buildings with large outdoor areas including flowering wild plants. Crop plants other than those placed in the mesocosms were not in the foraging range for bumblebees. While we can exclude exposure to pesticides outside the mesocosms by crop plants, we cannot exclude exposure from pesticide use in gardens, which, however is lower compared with exposure in agricultural areas (Nicholls et al. 2018; Siviter et al. 2023). All colonies were checked weekly to detect dead colonies indicated by a dead queen or a large number of dead workers. One colony died two weeks after the experiment had started.

Bumblebee observations were conducted between 9:00 and 16:00, from the 4th of June until the 3rd of July by one observer, who was blinded to the plant treatments. Observations were conducted during different weather conditions, which were recorded at the beginning of each observation session. Temperature was measured using a mobile weather station attached to one of the mesocosms, wind force was estimated according to the WMO classification of the Beaufort scale (WMO – World Meteorological Organization 2019) and proportion of cloud cover was estimated visually. Temperature during observations varied between 11 and 30 °C with a mean of 19 °C and wind speed between 1 and 5 Beaufort but 2–3 Beaufort most often estimated. Cloud cover varied between 0 and 100% with a mean of 45%. It did not rain during our observations.

The foraging activity of bumblebees was recorded hourly, in a randomized order across mesocosms. The random order was computed daily using R 3.1.1 (function ‘sample’-; package: base) (R Core Team 2015). The number of foraging individuals on each of the two sets of plants in the mesocosms was counted immediately when approaching a mesocosm. A bee was considered to be actively foraging when entering the corolla of flowers with its proboscis, drinking from the nectar (Eickwort and Ginsberg 1980) or flying between flowers. Nectar robbing was excluded from the analysis but results did not differ qualitatively when nectar robbing was included (GLM: z = −3.668; *P* < 0.001). Nectar robbing was defined according to Inouye (1980) as bees biting a hole into the corolla and drinking from the nectary directly instead of visiting the flower legitimately via the stigma entrance.

We observed 0–25 workers in each cage, varying between different times of day and in addition, the experiment was run over 30 days and hence, several generations of workers could have been observed within the cages. This is why we conclude that different bee individuals were foraging within the cages at each point of time of the observations.

### Statistical analysis

All analyses were done in R 4.0.3 (R Core Team 2022) using generalized linear mixed and generalized linear models ’glmmTMB’-function; package glmmTMB; (Brooks et al. 2017) to test treatment effects (treated versus control).

Glucosinolate levels were compared between clothianidin-treated vs. control plants using Gaussian models with treatment as a predictor. Glucosinolate levels were compared in flowers and leaves for the total sum of glucosinolates and individually for each of the detected compounds. Models for the content of Gluconapoleiferin showed strong differences in residual variances between treatment levels, which could not be handled with the glmmTMB models and hence the non-parametric exact Wilcox-Mann-Whitney test was used (’wilcox.exact’-function; package: exactRankTests) (Hothorn and Hornik 2021). Normal distribution of residuals was assumed for all analyses, except for the analysis of the contents of Glucobrassicanapin and Gluconapoleiferin in flowers, which we assumed to be Gamma-distributed, as well as the contents of Glucobrassicin and Gluconapin in leaves, where we assumed a Tweedie distribution.

Flower variables (days until flowering, floral display, number of flowers) and flower resources (pollen, nectar) were compared between clothianidin-treated and control plants on data aggregated by calculating mean values per plant, using Gaussian models with treatment as a fixed effect.

The foraging preference of bumblebees in relation to plant treatment was calculated as the proportion of bees feeding on clothianidin-treated vs. control plants for each mesocosm and tested using a beta distribution intercept-only model. We modeled the full data set including observations for several times a day (predictor: treatment; random: mesocosm id and observation day) and for observations aggregated per day (predictor: treatment; random: mesocosm id) using generalized mixed models, but both models had strong correlations between random effects (singularity) or could not estimate random effects and also showed autocorrelation. To solve this, data were aggregated across cages (one value per cage) and modeled using a beta-family generalized linear model. We reran the analyses excluding data from the mesocosm where the bumblebee colony died to be sure that this did not influence the results. Excluding this cage had no qualitative impact on the results.

Distributional assumptions (Binomial, Gaussian, Gamma, Tweedie) and homogeneous variance of residuals were tested for global and final models using simulated residuals ’simulateResiduals’-function; package DHARMa; (Hartig 2018). When necessary, it was accounted for different variances between treatment levels (’dispformula’-function; package: glmmTMB) (Brooks et al. 2017). In addition, the simulated residuals were used to check those models for zero-inflation ’testZeroInflation’-function; package DHARMa; (Hartig 2018) and under/overdispersion ’testDispersion’-function; package DHARMa; (Hartig 2018). *P*-values were obtained from the ’summary’-function for the preference of bumblebees (z-test) and from likelihood-ratio tests for all other variables.

## Results

**Fig. 1:**
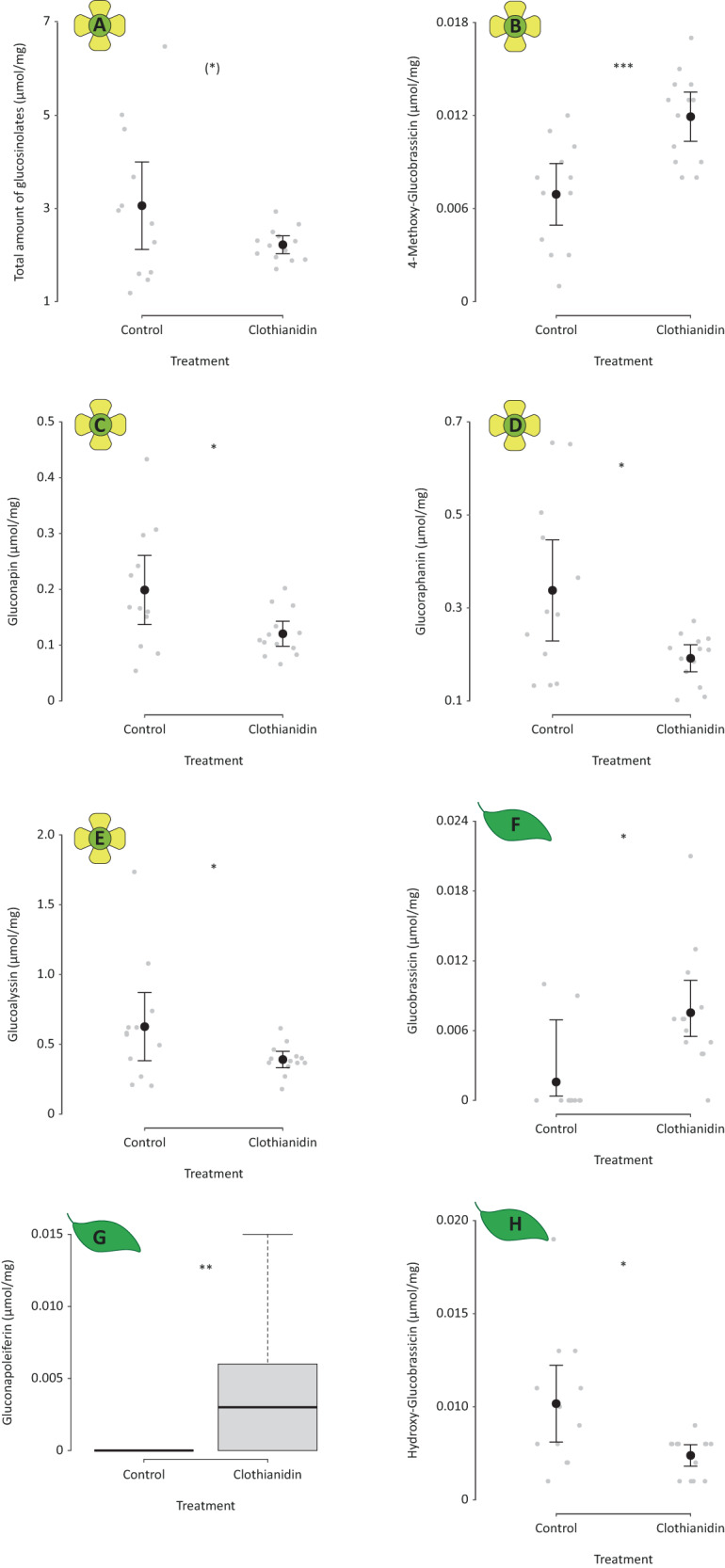
Glucosinolates in flowers and leaves differing between clothianidin-treated and control plants. (**A**) Total content of glucosinolates and content of the glucosinolates (**B**) 4-Methoxy-Glucobrassicin, (**C**) Glocunapoleiferin, (**D**) Gluconapin and (**E**) Glucoraphanin in flowers of clothianidin-treated and control oilseed rape plants, separately. Content of the glucosinolates (**F**) Glucobrassicin, (**G**) Gluconapoleiferin and (**H**) Hydroxy-Glucobrassicin in leaves of clothianidin-treated and control oilseed rape plants. Black points display mean values and error bars ± 95 % confidence intervals, obtained from model estimates; gray points display raw data. Lines in boxplots display the median, boxes upper and lower quartiles and whiskers minimum and maximum. (*)*P* < 0.1, *P < 0.01, **P < 0.001, ***P < 0.0001

The overall amount of glucosinolates in flowers was marginally, but not significantly, higher in control compared with clothianidin-treated plants (GLM: *X*^*2*^ = 2.926, *P* = 0.087; Fig. 1A). Broken down into individual substances, we found that flowers of treated plants had higher concentration of the indolic glucosinolate 4-Methoxy-Glucobrassicin (GLM: *X*^*2*^ = 12.286, *P* < 0.001, Fig. 1B) and lower concentrations of the aliphatic glucosinolates Gluconapin (GLM: *X*^*2*^ = 5.070; *P* = 0.024; Fig. 1C) and Glucoraphanin (GLM: *X*^*2*^ = 5.762; *P* = 0.016; Fig. 1D). The aliphatic glucosinolate Glucoalyssin was marginally, but not significantly, lower in the clothianidin treatment (GLM: *X*^*2*^ = 3.317, *P* = 0.069, Fig. 1E). Concentrations of the other six glucosinolates detected in flowers did not differ significantly between treatments (Supplementary Material Table S1). Also the leaves of treated plants showed changes in three of the nine detected glucosinolate compounds. Leaves of clothianidin-treated plants had higher concentrations of the indolic Glucobrassicin (GLM: *X*^*2*^ = 6.524; *P* = 0.011; Fig. 1F) and the aliphatic Gluconapoleiferin was only found in the leaves of treated plants (Exact Wilcox: W = 30; *P* = 0.002; Fig. 1G). In contrast, the indolic Hydroxy-Glucobrassicin had higher concentrations in control plants (GLM: *X*^*2*^ = 5.762; *P* = 0.016; Fig. 1H). Glucoalyssin was not found in leaves, and the other six glucosinolates, as well as the total concentration of glucosinolates, did not differ significantly between treatments (Supplementary Material Table S1).

The start of flowering, flower size, number of flowers, and amount of nectar and pollen produced were similar between treatments (Supplementary Material Table S2).

**Fig. 2:**
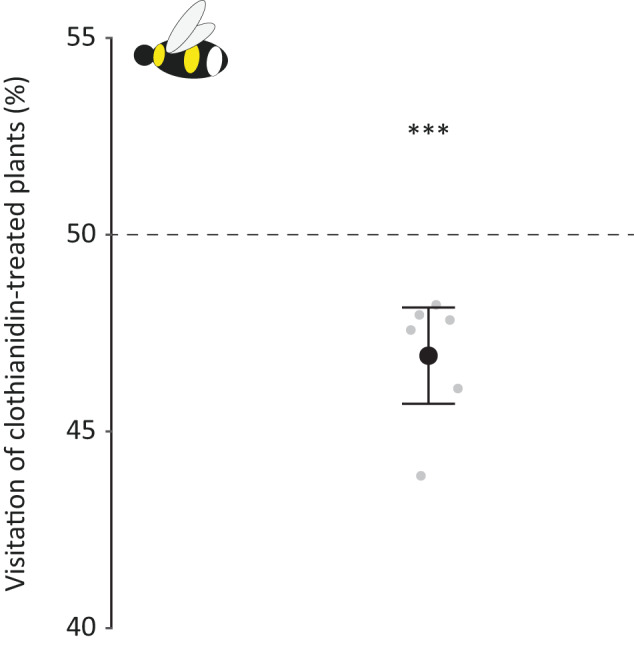
Flower visitation of *B. terrestris* on clothianidin-treated plants. Black point displays mean value and error bar ±95% confidence interval, obtained from model estimates; gray points display raw data. Dashed line indicates 50% level of visitation of clothianidin-treated plants. (*)*P* < 0.1, *P < 0.01, **P < 0.001, ***P < 0.0001

Bumblebees were less likely to forage on clothianidin-treated plants compared to control plants (GLM: z = −3.578; *P* < 0.001; Fig. 2), however, although significant, this effect was very small with a mean of 47.2 % and a range of 43.9–48.2% among the different mesocosms.

## Discussion

Treated plants had altered glucosinolate profiles but we did not observe any differences in flower number, time of flowering, size or resource provisioning. Nevertheless, bumblebees showed a small preference for control plants, suggesting that they detected and were influenced in their foraging decisions by changes in glucosinolate profiles.

Based on previous findings, we suggest that changes in the glucosinolate profile of oilseed rape plants were caused by the treatment with the neonicotinoid clothianidin. Analogous to herbivory, neonicotinoids can induce stress responses in plants, resulting in increased levels of the phytohormone salicylic acid (Ford et al. 2010), which in turn can alter glucosinolate profiles in oilseed rape (Kiddle et al. 1994). Similar to what has mostly been found in response to herbivory (Textor and Gershenzon 2009), we showed that clothianidin treatment mainly led to an increase in indolic glucosinolates (flowers: 4-Methoxy-Brassicin; leaves: glucobrassicin), with strongest effects on the induction of 4-Methoxy-Brassicin in our study system. However, we also found reduced levels of one indolic glucosinolate (Hydroxy-Glucobrassicin) in response to the clothianidin-treatment. This contrasts to herbivory studies, where indolic glucosinolates seldomly decrease and Hydroxy-Glucobrassicin is often one of the most increasing glucosinolates (Textor and Gershenzon 2009). Whereas herbivory can have a variety of different responses in terms of induction of glucosinolates (Textor and Gershenzon 2009), clothianidin, had previously only been shown to affect the salicylic acid pathway (Ford et al. 2010). Besides that, the induction of glucosinolates by the salicylic acid pathway can be weaker compared with another pathway based on jasmonic acid (Textor and Gershenzon 2009; Wiesner et al. 2013), salicylic acid can also antagonize the jasmonic acid pathway (Textor and Gershenzon 2009). Hence, complex interactions may have resulted in a decrease of Hydroxy-Glucobrassicin in response to clothianidin treatment, but this mechanism needs to be studied in more focused experiments. For aliphatic glocusinolates, responses to herbivory can vary largely with both increases (though to a lower extent than indolic glucosinolates) and decreases (Textor and Gershenzon 2009) being found, similar to findings of this study. As in previous studies (Textor and Gershenzon 2009), not all (indolic or aliphatic) glucosinolates were affected.

An intriguing question is by which mechanism foraging preferences are affected. Neonicotinoids are not volatile (Bonmatin et al. 2015) and bumblebees are thought to perceive neonicotinoids as tasteless and odorless (Muth et al. 2020). Yet there is some evidence that bumblebees prefer sugar solution with neonicotinoids (Kessler et al. 2015, Arce et al. 2018; but see Muth et al. 2020). In contrast, we found that bumblebees foraged slightly but significantly less on flowers on clothianidin-treated plants. Previous studies found that the costs related to an activated plant defense mechanism can affect plant vigor, growth, and development (Strauss et al. 2002; Kessler and Chautá 2020), but we did not find treatment effects on flower phenology, development, size or resource quantity. This might be due to induction of plant defense by clothianidin differing from inductions caused by herbivory, similar to the difference between chemically induced plant defense versus plant defenses induced by herbivory (Cipollini and Sipe 2001) or to differences found between herbivore species (Rusman et al. 2019). Another candidate explanation is an effect of altered glucosinolate profiles we found to be induced by the clothianidin treatment. Most information about effects of glucosinolates in plant preference relates to herbivores. Whereas glucosinolates can be attractant to specialized herbivores, they often repell (and are sometimes even toxic for) generalized insect herbivores (reviewed by Wittstock et al. 2003, Textor and Gershenzon 2009). Knauer and Schiestl (2017) found that *B. napa* plants that had increased levels of the glucosinolate Glucobrassicanapin caused by foliar herbivory tended to have reduced visitation by bumblebees, but this effect could also have been related to indirect effects such as delayed flowering or a change in volatile organic compounds (discussed below). To our knowledge, the only available test whether glucosinolates have a direct effect on foraging preference of bees was conducted by testing consumption of sugar solutions containing different concentrations of the glucosinolates Scopolamine, Amygdalin and Sinigrin by bumblebees (Sculfort et al. 2021). However, results were difficult to interpret, because both high and low but not intermediate concentrations of Amygdalin and Sinigrin repelled bees, whereas Scopolamine only had attractant effects (Sculfort et al. 2021). In addition we cannot exclude that additonal plant parameters not measured by us could be the causative agent. Herbivory can, for instance, alter the emission of floral volatile compounds in flowers as well as lead to higher sugar contents in nectar (Bruinsma et al. 2014; Rusman et al. 2019), but these effects can differ in relation to herbivore species (Rusman et al. 2019). In addition, effects on pollinator visitation from herbivory-realted changes in plants can differ between pollinator species as well as can result in preference, repellence or even be absent (Bruinsma et al. 2014; Rusman et al. 2019). Hence, the mechanism behind any difference in preference in our study can be complex and cannot be revealed with our study system and will have to be investigated in more detail.

We cannot rule out an effect of the treatment of seeds with the fungicide iprodione, but iprodiose was used in both of our treatments and any effect therefore needs to be caused by an interaction with the treatment effect. Iprodione is used against fungal diseases of roots and stem roots (Pohanish 2015) and was used to prevent fungal diseases on our plants in the greenhouse, which can affect glucosinolate production in oilsee rape (Li et al. 1999). It has been considered to be only locally systemic (Pohanish 2015), why it is unlikely that it occurred in nectar/pollen of our plants. However, iprodiane has been found in nectar and pollen sampled by honeybees (Zioga et al. 2020), although this may not originate from residues resulting from seed coating but instead from apical applications. In more general terms, we performed our study under specific circumstances, and only future research can disentangle how interactions between different plant protection products might result in plant physiological changes.

Our mesocosm experiment may have implications for foraging by wild bumblebees. Although we found that bumblebees preferred to forage on untreated plants, this difference in preference may be too small to be consequential when other factors determine foraging preferences. For example, under fully realistic field conditions bee preferences may also be overshadowed by neonicotinoids affecting herbivory and thus plant vigour (Lindström et al. 2018), as well as effects of distance to crops on foraging preferences (Dramstad et al. 2003). Results might also differ between crop species, varieties and pesticides because of variation in how seed coating affects the profile and availability of glucosinolates (Fahey et al. 2001) or other secondary plant products.

If preference develops over time (Arce et al. 2018), our study may however, have underestimated effect sizes since most bees foraged outside of the mesocosm (as allowed by our t-shaped colony entrance). Thus, while seed coating with clothianidin is currently banned for flowering plants in the EU, our results may have implications for exemptions from the ban as well as for parts of the world where clothianidin is still used (Bonmatin et al. 2015; European Commission 2021). Furthermore, other substances used for seed coatings such as Flupryidafurone and Sulfoxaflor are also systemic in plants and with the same mode of action in insects, affecting the nicotinic acetylcholine receptor (Zhu et al. 2011; Nauen et al. 2015) potentially influence plant attractiveness similarly, but this will have to be tested in further studies.

## Conclusions

The treatment of plants with clothianidin altered the production of glucosinolates and hence induced changes in plant metabolism but not other pollinator-relevant traits such as flower phenology, size or resource provision in oilseed rape plants. We cannot prove that lower visitation of bees on clothianidin-treated plants was a result of changes in glucosinolate profiles and it is possible that bees perceived other or additional neonicotinoid-induced changes in plant metabolism. Although our results suggest that bees show a slight preference for untreated oilseed rape, it is important to note that the difference was small and possibly not relevant under field-realistic conditions where distances between treated and untreated oilseed rape is usually much larger. Nevertheless, our results contribute to understanding of how neonicotinoids impact pollinator foraging but they also show that more research is needed, for instance based on individual observations or a more realistic setup including herbivores, to reveal potentially complex mechanisms between crop exposure to neonicotinoids and pollinator foraging choices.

### Supplementary information

Supplementary Information
