# Supplementary material for: Seed treatment with clothianidin induces changes in plant metabolism and alters pollinator foraging preferences
Source: Ecotoxicology. 2023 Dec 7;32(10):1247–56. doi: 10.1007/s10646-023-02720-0 (PMC10724316; doi:10.1007/s10646-023-02720-0)
Supplement: Supplementary file 1 — Supplementary Information [file 10646_2023_2720_MOESM1_ESM.docx]

Supplementary material for:

Seed treatment with clothianidin induces changes in plant metabolism and alters pollinator foraging preference

Björn K. Klatt, Annemarie Wurz, Lina Herbertsson, Maj Rundlöf, Glenn P. Svensson, Jürgen Kuhn, Sofie Wessling, Bernardo de La Vega, Teja Tscharntke, Yann Clough & Henrik G. Smith

**Supplementary material Fig. S1: (A)** Set up of mesocosms. Rapeseed plants were either grown from seeds coated with Elado® and Rovrl® (clothianidin-treated plants, yellow circles with ‘+‘) or only Rovral® (control-plants, yellow circles with ‘–‘). Orange boxes display colonies of *B. terrestris*, black vertical lines additional mesh constructions that were installed to limit edge effects. **(B)** Mesocosm set up in detail. **(C)** Bumblebee colony setup in detail. Bees were allowed to forage inside and outside the mesocosms through a t-shaped tube connecting the entrance of the colony with the inside of the mesocosm and the outside.

**Supplementary material Table S1:** Differences in glucosinolate levels between clothianidin-treated and control plants. Statistically significant differences (P <0.050) are marked in **bold** and marginally significant differences (P <0.100) are marked in *italics*. CI = 95 % confidence intervals, C = control plants, T = treated plants.

| Compound | Flowers |  |  |  | Leaves |  |  |  |  |
| --- | --- | --- | --- | --- | --- | --- | --- | --- | --- |
|  | *X^2^* - value | P - value | mean | lower/upper CI | *X^2^* - value | P - value | mean | lower/upper CI |  |
|  |  |  |  |  |  |  |  |  |  |
|  |  |  |  |  |  |  |  |  |  |
| Total amount | *2.926* | *0.087* | C: 3.061 | C: 2.120/4.000 | 0.889 | 0.346 | C: 0.108 | C: 0.097/0.119 |  |
|  |  |  | T: 2.222 | T: 2.032/2.413 |  |  | T: 0.117 | T: 0.101/0.132 |  |
| 4-Methoxy-Glucobrassicin | **12.286** | **< 0.001** | C: 0.007  T: 0.012 | C: 0.005/0.009  T: 0.010/0.014 | 0.526 | 0.468 | C: 0.002  T: 0.002 | C: 0.001/0.002  T: 0.001/0.002 |  |
| Glucoalyssin | *3.317* | *0.069* | C: 0.627  T: 0.391 | C: 0.383/0.871  T: 0.332/0.450 | *Not found in leaves* | |  |  |  |
| Glucobrassicanapin | 1.700 | 0.192 | C: 0.063  T: 0.047 | C: 0.047/0.095  T: 0.036/0.069 | 0.391 | 0.532 | C: 0.019  T: 0.021 | C: 0.013/0.025  T: 0.016/0.028 |  |
| Glucobrassicin | 0.844 | 0.358 | C: 0.264  T: 0.232 | C: 0.201/0.327  T: 0.202/0.263 | **6.524** | **0.011** | C: 0.002  T: 0.008 | C: 0.0004/0.007  T: 0.005/0.011 |  |
| Gluconapin | **5.070** | **0.024** | C: 0.199  T: 0.120 | C: 0.137/0.261  T: 0.098/0.143 | 0.644 | 0.422 | C: 0.006  T: 0.009 | C: 0.003/0.014  T: 0.006/0.013 |  |
| **Supplementary material Table S1:** continued. | | | | | | | | |  |
| Compound | Flowers |  |  |  | Leaves |  |  |  |  |
|  | *X^2^* - value | P - value | mean | lower/upper CI | *X^2^* - value | P - value | mean | lower/upper CI |  |
|  |  |  |  |  |  |  |  |  |  |
|  |  |  |  |  |  |  |  |  |  |
| Gluconapoleiferin | 0.882 | 0.348 | C: 0.026  T: 0.031 | C: 0.021/0.036  T: 0.025/0.042 |  | **0.002*** | C: 0.000  T: 0.004 | C: 0.000  T: 0.004 |  |
| Glucoraphanin | **5.762** | **0.016** | C: 0.338  T: 0.192 | C: 0.229/0.446  T: 0.163/0.221 | 0.468 | 0.494 | C: 0.028  T: 0.024 | C: 0.018/0.039  T: 0.016/0.032 |  |
| Glucotropaeolin | 2.561 | 0.110 | C: 0.010  T: 0.012 | C: 0.007/0.012  T: 0.010/0.015 | 1.417 | 0.234 | C: 0.004  T: 0.004 | C: 0.004/0.005  T: 0.004/0.004 |  |
| Hydroxy-Glucobrassicin | 0.109 | 0.751 | C: 0.044  T: 0.046 | C: 0.032/0.056  T: 0.039/0.053 | 5.762 | **0.016** | C: 0.010  T: 0.007 | C: 0.008/0.012  T: 0.007/0.008 |  |
| Progoitrin | 1.851 | 0.174 | C: 1.484  T: 1.138 | C: 0.986/1.982  T: 1.038/1.238 | 0.373 | 0.541 | C: 0.036  T: 0.038 | C: 0.034/0.038  T: 0.033/0.042 |  |

*Gluconapoleiferin was not found in the leaves of control plants; wilcox exact test was used for analysis

and standard deviation to show variance of treatments.

**Supplementary material Table S2:** Differences in flowering and floral resource between clothianidin-treated and control plants. CI = 95 % confidence intervals.

| Trait | *X^2^* - value | P - value | mean | lower/upper CI |  |
| --- | --- | --- | --- | --- | --- |
|  |  |  |  |  |  |
| Flowers produced | 0.164 | 0.685 | C: 64.667  T: 62.600 | C: 56.917/72.416  T: 54.111/71.089 |  |
| Floral display | 0.386 | 0.534 | C: 19.415  T: 19.241 | C: 18.992/19.838  T: 18.778/19.704 |  |
| Days until flowering | 0.007 | 0.934 | C: 46.167  T: 46.000 | C: 43.106/49.228  T: 42.647/49.353 |  |
| Pollen produced | 0.159 | 0.690 | C: 0.003  T: 0.003 | C: 0.0028/0.0038  T: 0.0029/0.0034 |  |
| Nectar produced | 0.317 | 0.573 | C: 0.111  T: 0.098 | C: 0.074/0.148  T: 0.057/0.138 |  |
